# Robotic Grasping of Unknown Objects Based on Deep Learning-Based Feature Detection

**DOI:** 10.3390/s24154861

**Published:** 2024-07-26

**Authors:** Kai Sherng Khor, Chao Liu, Chien Chern Cheah

**Affiliations:** 1 School of Electrical and Electronics Engineering, Nanyang Technological University, Singapore 639798, Singapore; khor0054@e.ntu.edu.sg; 2 Department of Robotics, Laboratory of Computer Science, Robotics and Microelectronics of Montpellier, Centre National de la Recherche Scientifique, University of Montpellier, 161 Rue Ada, 34095 Montpellier, France; chao.liu@cnrs.fr

**Keywords:** robotics, robotic grasping, unknown objects

## Abstract

In recent years, the integration of deep learning into robotic grasping algorithms has led to significant advancements in this field. However, one of the challenges faced by many existing deep learning-based grasping algorithms is their reliance on extensive training data, which makes them less effective when encountering unknown objects not present in the training dataset. This paper presents a simple and effective grasping algorithm that addresses this challenge through the utilization of a deep learning-based object detector, focusing on oriented detection of key features shared among most objects, namely straight edges and corners. By integrating these features with information obtained through image segmentation, the proposed algorithm can logically deduce a grasping pose without being limited by the size of the training dataset. Experimental results on actual robotic grasping of unknown objects over 400 trials show that the proposed method can achieve a higher grasp success rate of 98.25% compared to existing methods.

## 1. Introduction

Grasping has remained a significant focus of research in robotics for decades, enabling robots to interact proficiently with objects in the real world. Humans, upon looking at an object, even for the first time, instinctively know where to pick it up from. This, coupled with the incredible dexterity of the human hand, makes any grasping attempt almost always a success. However, this is a highly challenging task for current robot systems, as robots do not have such instinct nor dexterity [1].

Modern-day robots are highly advanced; they can be programmed to perform highly complex movements with high accuracy and precision. However, these programs tend to be specific to a certain object or environment. A change in any of these circumstances renders these programs unreliable and a new program has to be designed. This lack of flexibility in robot grasping is a challenging problem [2,3]. The rise of deep learning algorithms has provided a possible solution to this problem [4] by introducing data-driven techniques. Through the interactions between sensors and the environment, grasp pose generations can be achieved by imitating human grasping strategies [5]. Integrating Deep Convolutional Neural Networks (CNNs) into grasping algorithms allows robots to deal with changes in the environment more readily. Modern data-driven techniques for robotic grasping involve teaching a robot with large amounts of data instead of training with physical object models [6]. These training data usually contain many images of each object in various positions and orientations, and the grasping pose is manually labelled [7,8]. Once the training is completed, the robot can grasp these objects regardless of how they are placed. Significant past works in grasping such as those by Pinto et al. [9] and Levine et al. [10] approached the problem by amassing large quantities of data over an extended period of time, 50,000 and 800,000 datapoints, respectively. Many grasps are executed, and the results of those grasps are recorded in terms of success or failure. This allows the algorithm to learn what grasps are good. The resultant algorithm was effective in grasping specific objects. Another technique that has been explored is to use object detectors as part of the grasping algorithm. Modern object detectors are highly accurate and run considerably fast; a few iterations can run in a second, making it feasible for real-time usage. This is comparative to the early stages of CNN development, whereby the high computational cost and long run times per iteration make them unsuitable for real-time detection and grasping [11]. Kim et al. [12] have trained an object detector using the images and grasping points of two object classes from the Open Image Dataset (OID). The resultant model can pick up these two classes of objects successfully with around 70% precision. Huang et al. [13] approach grasping by integrating multi-agent deep reinforcement learning with an object detector. The resultant MA-TD3H algorithm has a very high success rate in grasping objects, but can only grasp the three objects that it is trained on, and it is untested on unknown objects outside of this training dataset. Some other studies find that using an object detector alone is too limited and approach the problem differently. Zhang et al. [14], Yang et al. [15], and Geng et al. [16] propose a two-stage cascaded algorithm by first using the object detector to identify and localize an object before using a second Grasp Generation CNN (GGCNN) to determine the grasp position. A more recent study by Li et al. [17] first utilizes an object recognition algorithm to recognize six different categories of objects, and then develops a GGCNN that is trained with grasp positions for each category. Upon encountering an object, the object recognition module determines the category that best matches it. The GGCNN then generates a grasp position for it. The approach sports an 86% grasp success rate. However, it was noted that these systems are highly unscalable due to the limitations of the training dataset and are unable to deal with objects outside of the training. Another trade-off of cascaded algorithms is the runtimes. Geng et al. [16] found that it ran up to 4 times slower than just having the object detector alone. To deal with this limitation, multiple studies have gone beyond just robotic grasping and delved into unknown object robotic grasping. This refers to objects that are not seen by the system during the training process. One of the earliest works specifically targeting unknown object grasping is by Saxena et al. [18]. In that work, 2500 datapoints were synthetically generated to train the algorithm and it predicts a grasp pose directly as a function of the images via triangulation of a grasping point, hence removing the need to be trained on any specific objects. The algorithm has a 87.5% success rate on a collection of eight unknown objects. Mahler et al. [19] developed Dex-Net 2.0 with a synthetic dataset of 6.7 million datapoints. The algorithm showed an 80% success rate in grasping a set of 10 unknown objects. Morrison et al. [20] developed a generative grasping convolutional neural network (GG-CNN) which directly generates a dense prediction of antipodal grasp poses and a quality measure for every pixel in an input depth image. They achieved a 94% success rate on a set of 12 unseen household items with a training size of 51,100 datapoints. Zhang et al. [21] tackle unknown object grasping by integrating visual information with tactile information as part of their data-driven algorithm. The trained model’s performance varies greatly with the unknown object experimented with, having an accuracy of between 40% and 92%. Coupled with the large training dataset of 23,600 datapoints, the algorithm does not seem to generalize well to all unknown objects. A more recent study by Eguiluz et al. [22] approaches the unknown object grasping problem from a different perspective. Data-driven techniques are not used and the algorithm falls back on a more classical grasping approach using contact forces. The robot first touches the unknown object to collect more information through tactile sensors, which are combined with the RGB-D image to form a valid grasp position. As the robot is not trained via any CNN to recognize any object, all objects are new to the robot and it has good grasp performance. However, this two-step approach is slower. It requires 0.5 s to generate a grasp position, which is considerably slower than many other visual-based data-driven grasping algorithms that can do the same in less than 0.1 s. As such, some approaches with increased complexity or combining contact forces can improve the performance when approaching unknown objects but have a significant trade-off in speed.

In a world with an infinite number of different objects, it is both inefficient and impossible to train the algorithms to recognize every object. As such, a purely data-driven approach for object grasping is not ideal. Therefore, it stands as a critical challenge in the literature of robotic grasping to develop an efficient method that will not require a large amount of training data but still can achieve a high success rate in actual grasping of unknown objects. To tackle this problem, a novel method is presented in this paper for robotic grasping by combining data-driven techniques with information obtained through image segmentation and logical deduction to propose a grasp pose for each unknown object. Instead of training a robot to recognize objects and their grasp positions, the algorithm is trained to identify common object features, namely straight edges and corners. Combining these with some basic object information that can be extracted from the image of an object, the algorithm can logically deduce a grasp position. This algorithm does not require the tedious labelling of grasp positions on different objects, neither does it need to identify what class of object it encounters. As these are object features or object parameters that are present in almost all objects, the robot is able to attempt to grasp it even if it is not part of the training dataset and without the need to know what the object is. The algorithm achieves higher performance in grasping unknown objects than existing methods without the need for large amounts of training data.

## 2. Methodology

When humans grasp an object, we generally grasp it between the fingers and thumb by applying force from opposite sides. In our grasp planning, flat surfaces of the objects are preferred and corners are avoided where possible. For circular objects without flat surfaces or corners, we grasp it along the diameter. The grasping point that is chosen also tends to be near the center of gravity of the object if possible. The center of gravity can usually be estimated quite accurately visually, as it tends to be around the center of the object. This paper takes inspiration from these observations in human grasping to develop a robotic grasping algorithm.

### 2.1. Architecture

A 2D image *X* of an object is the input and it passes through two concurrent processes: *feature extraction* and *parameter extraction*. The feature extraction process involves identifying two key features of the object in the image, namely a set of straight edges:

(1) $$E=\{E_{1},E_{2},\ldots ,E_{n}\},$$

and a set of corners:

(2) $$C=\{C_{1},C_{2},\ldots ,C_{m}\},$$

where *n* is the number of straight edges detected and *m* is the total number of corners detected.

**Figure 1:**
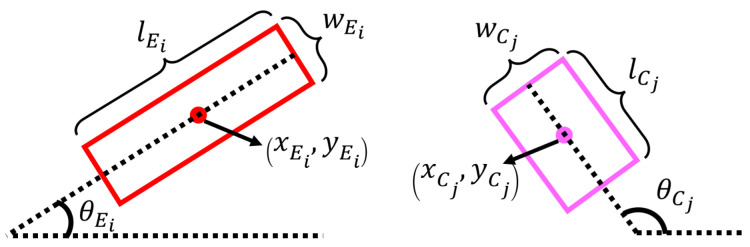
Illustration of bounding box parameters for straight edges $E_{i}$ and corners $C_{i}$.

To achieve this, an oriented object detector is trained based on images of objects that have their edges and corners labelled instead of the class of the object. An oriented object detector is used and each detected feature is represented by a bounding box. The coordinates of the center of a bounding box for each edge $E_{i}\in E$ are represented by $p_{E_{i}}=\left(x_{E_{i}},y_{E_{i}}\right)$. Each bounding box has a length $l_{E_{i}}$, a width $w_{E_{i}}$, and an angle $\theta_{E_{i}}$, where i=1,…,n. Similarly, for each detected corner $C_{j}\in C$, the coordinates of the center of each bounding box are $p_{C_{j}}=\left(x_{C_{j}},y_{C_{j}}\right)$, and it has a length $l_{C_{j}}$, a width $w_{C_{j}}$, and an angle $\theta_{C_{j}}$, where j=1,…,m. An illustration of the bounding box parameters is shown in Figure 1.

**Figure 2:**
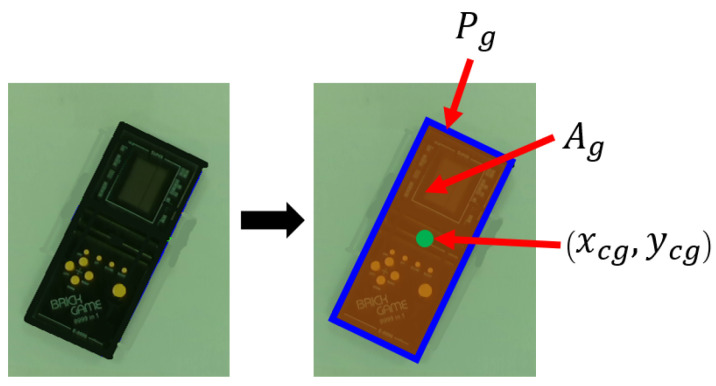
Illustration of geometric perimeter ($P_{g}$) in blue, geometric area $\left(A_{g}\right)$ in orange, and estimated geometric centroid $\left(x_{cg},y_{cg}\right)$ in green.

The object centroid $\left(x_{c},y_{c}\right)$, perimeter (*P*), and area (*A*) are also extracted. The parameter extraction process first uses an image segmentation algorithm to geometrically determine the perimeter ($P_{g}$) of the object. The area which is enclosed by the perimeter is then determined as $\left(A_{g}\right)$. The average of all the points on the perimeter also provides an estimated geometric centroid $\left(x_{cg},y_{cg}\right)$ of the object. An illustration is shown in Figure 2.

Next, all the detections from the feature extraction process are used to form a concave hull. This refers to the smallest geometric shape enclosing all points in a given set, which provides an abstract shape of the object and hence the estimated perimeter ($P_{e}$), area ($A_{e}$), and centroid $\left(x_{ce},y_{ce}\right)$ of the object. This abstract shape serves two important purposes. First, the parameters of the abstract shape are compared to the parameters obtained from image segmentation. From this comparison, the algorithm can determine if the features detected are a good or poor representation of the actual object. Secondly, it improves robustness of the algorithm. Should the parameter extraction fail to determine $P_{g}$ and hence $\left(x_{cg},y_{cg}\right)$ of the object due to unforeseen circumstances, $P_{e}$ and $\left(x_{ce},y_{ce}\right)$ can be used instead.

**Figure 3:**
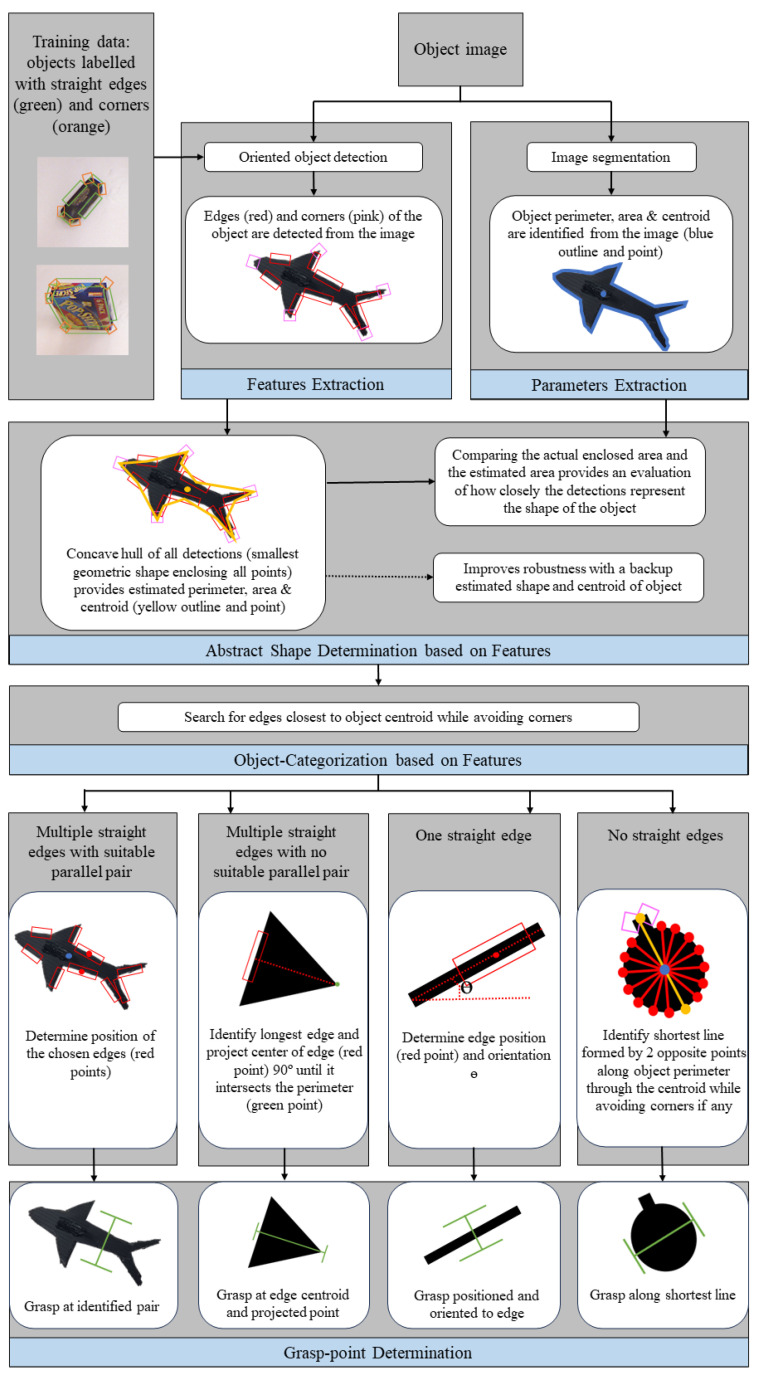
Overview: The input is the image of an object and it passes through two concurrent processes. The first is an oriented object detector trained on images of objects labelled with straight edges and corners which detects these same features in the image. The other step is image segmentation, which extracts the parameters (perimeter, area, and centroid) of the object. An abstract shape can be derived from the detected features and compared to the actual shape to evaluate the quality of the detections. The algorithm then searches for a pair of parallel straight edges as close to the object centroid as possible. Depending on the detections, the algorithm executes a different grasp strategy.

After the determination of the abstract shape of the object, the algorithm then first attempts to search for a pair of parallel edges closest to the object centroid. For objects with |E|=n≥2, the algorithm pairs each edge with another to find a suitable pair closest to the centroid. The grasp position is then determined from these two edges. If no suitable parallel pair of edges can be identified, the algorithm instead identifies the longest edge, as it is likely to be the most secure surface for grasping. The center of the longest edge is then projected 90° toward the other side of the object until it intersects the perimeter. The edge and this projected point provide the grasp position. If the distance between these two points is too far apart for grasping, the algorithm then moves on to the next longest edge and repeats the process until a suitable position is found. For objects with |E|=n=1 and small *A*, the object is likely small and thin, like a pen. The position and orientation of the edge gives the grasp position. If |E|=n=∅, the object could be very irregular and contain only detected corners (|C|=m≠∅) or be a circular object with no corners at all (|C|=m=∅). In such cases, the logic using straight edges is not possible and the algorithm employs a different strategy. A rotational search is performed about the object centroid and along the perimeter for two points that give the shortest grasp distance. If |C|≠∅, the points enclosed by the corner’s bounding box are avoided. An overview of the proposed algorithm is shown in Figure 3 and the key algorithms are presented in Section 2.8.

### 2.2. Feature Learning

**Figure 4:**
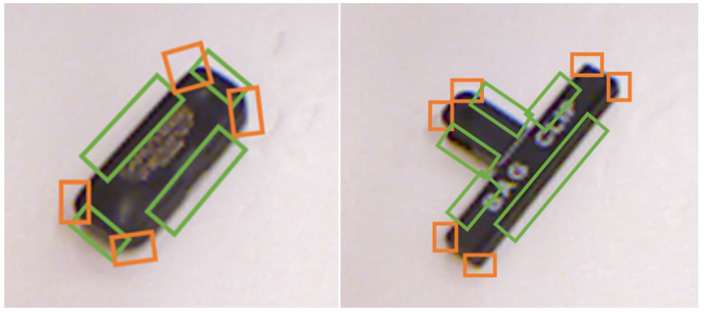
Labeled data for feature learning: examples of training data labelled for feature learning. Green bounding boxes represent edges and orange bounding boxes represent corners.

The basis for this algorithm lies in feature learning. By recognizing common features in objects instead of specific objects, the proposed algorithm possesses the flexibility to deal with a larger variety of situations. Figure 4 shows examples of some of the data used for feature learning. Note that curved corners are also labelled as corners to account for the facts that many real-world objects are deliberately manufactured to have rounded corners for safety purposes (for example, cell phones). Unlike other object recognition algorithms whose training data are labelled with many different classes, the feature learning in this algorithm is labelled with only two classes: edges and corners. It is important to note that the bounding boxes are oriented. It is vital for the algorithm to determine the orientation of these features, as it is used in later steps of the algorithm to determine a grasp position. After the training is complete, the detector is able to identify edges and corners from an image of an object.

**Figure 5:**
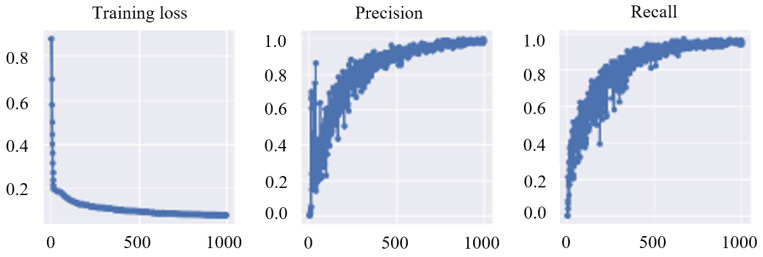
Training losses, precision, and recall of the feature learning.

In the proposed method, any oriented object detector based on deep learning can be used. Figure 5 shows the statistics of the training over 1000 epochs for 400 images using YOLOv5 OBB. The training loss decreases significantly over time and starts to plateau around 800 epochs. The precision and recall shows a high percentage of accurate predictions, with 98% correct predictions for both straight edges and corners on a clear background. These training results show the suitability of training the deep learning-based object detector to recognize these features and hence to develop a grasping algorithm from it.

### 2.3. Abstract Shape Determination and Image Segmentation

The abstract shape determination is achieved by creating a concave hull using the center points of all feature detections. That is, the abstract shape $S_{abstract}$ of the object is defined as:

(3) $$S_{abstract}=Conc\left(P_{E}\cup P_{C}\right)$$

where

(4) $$P_{E}=\left\{p_{E_{1}},p_{E_{2}},\ldots ,p_{E_{n}}\right\},$$

(5) $$P_{C}=\left\{p_{C_{1}},p_{C_{2}},\ldots ,p_{C_{m}}\right\}.$$

**Figure 6:**
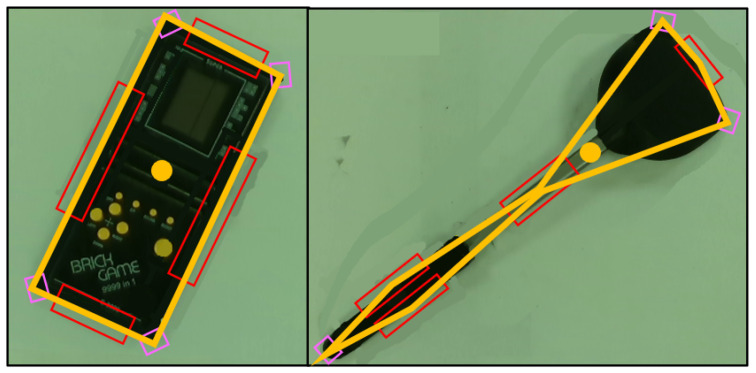
Feature detection and determination of abstract shape: The red boxes show the detected straight edges and the pink boxes show the detected corners. The yellow line shows the concave hull of all these detections, which provides the estimated perimeter and enclosed area of the object. The yellow circle shows the estimated centroid of the object derived from this concave hull.

This refers to the smallest geometrical shape formed which encloses the set of the feature points to give a general shape. Figure 6 shows the illustrations of the abstract shapes of two objects with the features determined. The abstract shape is the yellow line which gives the estimated perimeter $P_{e}$. The estimated area is $A_{e}$ and the estimated centroid $\left(x_{ce},y_{ce}\right)$ is represented by the yellow circle. If there are less than three detections such that |E∪C|<3, this step is bypassed.

**Figure 7:**
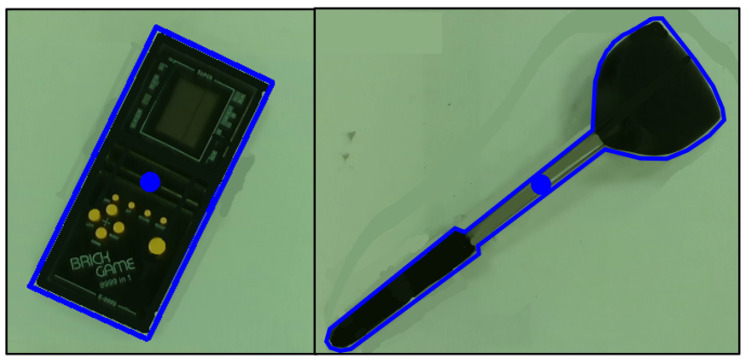
Image segmentation and parameter extraction: two objects with their geometric perimeter and centroid highlighted in blue.

An edge detection algorithm is also used to determine the borders of the object in the image *X*. This is the perimeter $P_{g}$, which is the blue line that outlines the object, as illustrated in Figure 7. The area enclosed by $P_{g}$ is $A_{g}$, and averaging all the points on $P_{g}$ gives the geometric centroid of the object $\left(x_{cg},y_{cg}\right)$ represented by the blue circle. Comparing $A_{g}$ and $A_{e}$ provides an idea of how closely the features represent the object. If $\left(x_{ce},y_{ce}\right)$ is also close to $\left(x_{cg},y_{cg}\right)$, it also shows its suitability in substituting $\left(x_{cg},y_{cg}\right)$ should image segmentation fail.

### 2.4. Determination of Grasp Position from Parallel Edges

**Figure 8:**
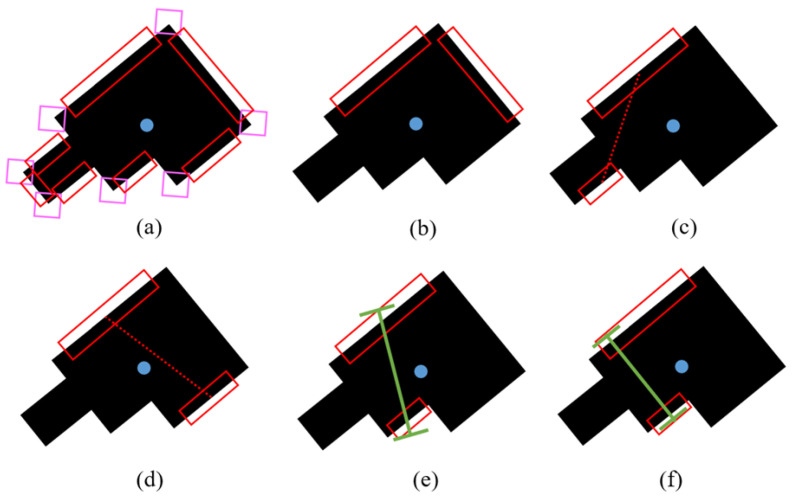
An object with multiple straight edges. (**a**) All detected features of the object. (**b**) Edges that have a large angle difference are not graspable. (**c**) Similarly, edges that are poorly aligned are poor grasp choices. To check for this, a line is drawn between 2 edges and its angle difference with the edges should be larger than $\theta_{diff\_min}$. (**d**) A pair can be parallel and be properly aligned, but if the distance between them is too far apart, it is not within gripper reach. (**e**) Another more suitable pair is chosen. If the differences in length of the bounding boxes are not accounted for and the grasp position is derived only by the middle of the bounding boxes, the grasp position is poorly oriented. (**f**) After optimization, a better grasp position is achieved.

With the key features and parameters of the object (straight edges, corners, perimeter, area, and centroid) extracted, the algorithm can then proceed to derive a grasp position. The algorithm determines a grasp position by identifying the two points where a two-fingered robot gripper should contact. From the features detected, the algorithm starts with a search for parallel straight edges closest to the object centroid to grasp while avoiding the corners. Figure 8a illustrates an example of an object with multiple detected features. For each $E_{i}\in E$, the distance $D_{i}$ from the object centroid $\left(x_{c},y_{c}\right)$ is calculated as:

(6) $$D_{i}=\sqrt{{\left(x_{c}-x_{E_{i}}\right)}^{2}+{\left(y_{c}-y_{E_{i}}\right)}^{2})}$$

All $E_{i}\in E$ are then sorted in ascending order of $D_{i}$. Starting with i=1, $E_{i}$ and $E_{i+1}$ are compared to find a pair that is parallel by calculating the difference in angle Δθ between the two edges:

(7) $$\Delta \theta \;=\;|\theta_{E_{i}}-\theta_{E_{i+1}}|\;\leq \theta_{max}$$

where the initial threshold value $\theta_{max}$ is user-defined. For example, an initial $\theta_{max}=10$° can be considered as close to parallel. Figure 8b illustrates the comparison between two edges. Δθ between these two edges is close to 90° and hence is larger than the threshold. As such, this pair is not chosen.

Following this step, a series of checks ensure that the pair is properly aligned for grasping. This is accomplished by drawing a line between the center of the two detected edges $x_{E_{i}},y_{E_{i}}$ and $x_{E_{i+1}},y_{E_{i+1}}$. The acute angle $\theta_{line}$ formed by the line and one parallel edge is then calculated. The line should have an angle difference with the angles of detections greater than a user-defined threshold $\theta_{diff\_min}$:

(8) $$|\theta_{line}-\theta_{E_{i}}|\;\geq \theta_{diff\_min}$$

for instance, $\theta_{diff\_min}=60$°. A poorly aligned pair of features, as depicted in Figure 8c, will likely be difficult to grasp. This pair is not selected, as the angle difference is less than 60° from the angles of the detections and is deemed unsuitable.

The next check ensures that the distance between the two identified edges is not larger than the maximum opening of the gripper. This maximum opening $d_{max}$ in pixels can be calculated as follows:

(9) $$d_{max}=m\times \frac{f}{d}$$

where *m* is the measured physical maximum opening of the gripper in cm, *f* is the focal distance of the camera in pixels, and *d* is the measured physical distance of the camera from the workspace in cm. Figure 8d shows a shortlisted feature pair for grasping. Although they have a small Δθ and are properly aligned, the distance between them is larger than $d_{max}$ and hence is not graspable. As such, another pair with distance less than $d_{max}$ is chosen, as illustrated in Figure 8e. After choosing a suitable pair of edges, some optimization is performed to account for the difference in lengths of the edges detected. If the algorithm determines the grasp position from the center of the bounding box without accounting for the differences in length, it results in a grasp position with a poor orientation, as illustrated in Figure 8e. It can be observed that the robot fingers do not have to be in the center of the detected bounding box; it can be anywhere along the length of the bounding box. With optimization, a grasping position that is better oriented can be generated, as shown in Figure 8f.

**Figure 9:**
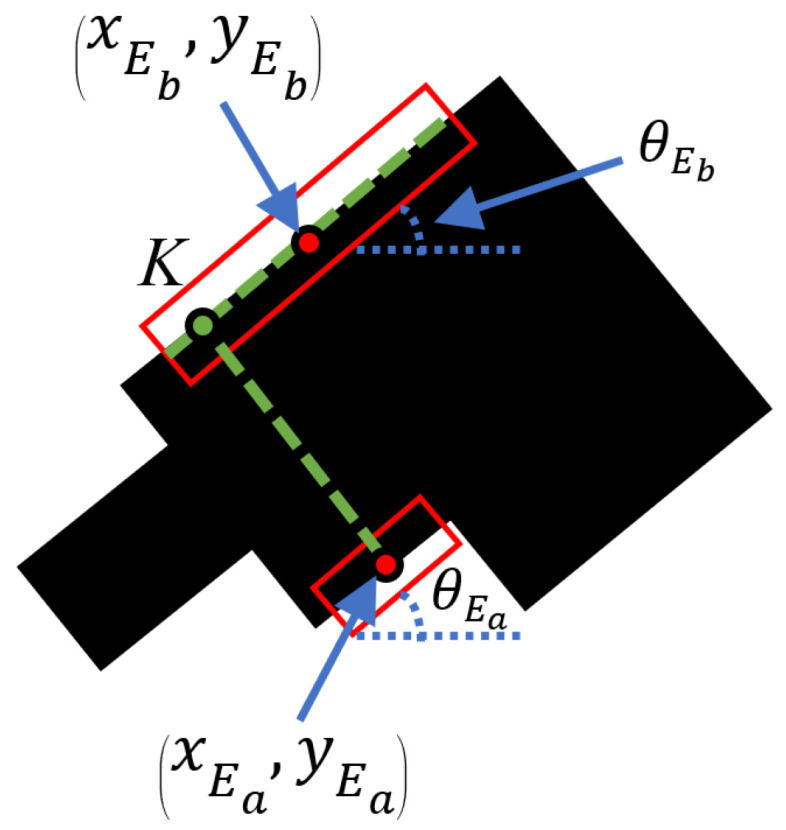
A line parallel to longer bounding box $E_{b}$ and a line perpendicular to shorter bounding box $E_{a}$ can be formed from bounding box information. The intersection of these 2 lines at point *K* gives the projection of the center of $E_{a}$ along the length of $E_{b}$. $\left(x_{E_{a}},y_{E_{a}}\right)$ and point *K* provide an optimized grasp position.

Optimization is achieved by projecting the center of the shorter bounding box on the longer bounding box. Let $E_{a}$ represent the shorter edge of length $l_{E_{a}}$ with center $\left(x_{E_{a}},y_{E_{a}}\right)$ and orientation $\theta_{E_{a}}$, and $E_{b}$ represent the longer edge of length $l_{E_{b}}$ with center $\left(x_{E_{b}},y_{E_{b}}\right)$ and orientation $\theta_{E_{b}}$. With $\left(x_{E_{b}},y_{E_{b}}\right)$ and $\theta_{E_{b}}$, a line parallel to $E_{b}$ can be formed. Similarly, with $\left(x_{E_{a}},y_{E_{a}}\right)$ and $\theta_{E_{a}}$, a line perpendicular to $E_{a}$ can be formed. This line represents the projection of $\left(x_{E_{a}},y_{E_{a}}\right)$ towards $E_{b}$. The intersection of these two lines gives point *K*, which is the resultant point from the projection of $\left(x_{E_{a}},y_{E_{a}}\right)$ along the length of $E_{b}$. Hence, the two points of contact for the gripper are $\left(x_{E_{a}},y_{E_{a}}\right)$ and point *K* to achieve an optimized grasping position. This idea of optimization is illustrated in Figure 9.

**Figure 10:**
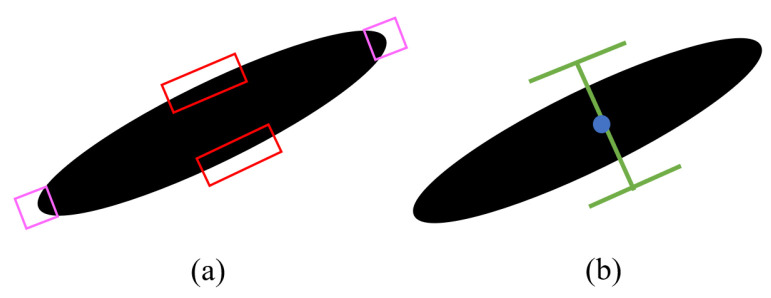
(**a**) For elongated ovals, the long sides are detected as straight edges and the pointed sides are detected as corners. (**b**) The algorithm can generate a grasp position based on the parallel edge logic.

It is observed that the parallel edge logic also works on non-angular shapes, such as long ovals. The pointed sides of the oval are detected as corners and the elongated sides detected as edges, as illustrated in Figure 10a. Hence, the algorithm is able to generate a grasp position from these two edges, as shown in Figure 10b.

### 2.5. Moving Threshold of Edges

**Figure 11:**
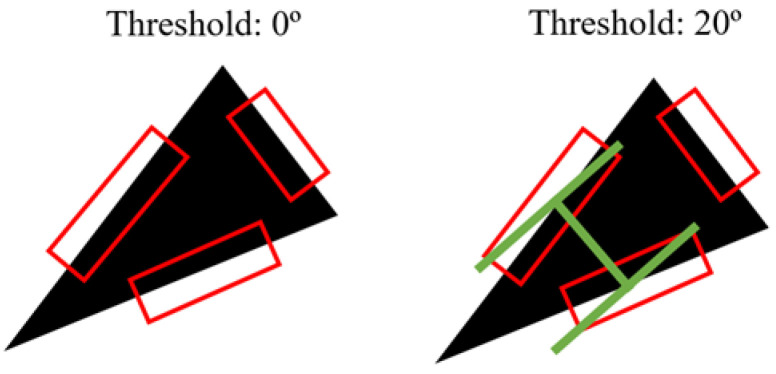
Object without parallel straight edges: A triangle is shown with 3 detected straight edges. When the threshold is set at 0°, there is no suitable combination of parallel pairs. When the threshold is loosened to 20°, a pair is chosen and the grasp position is established.

The edges need not be perfectly parallel to be grasped. It is observed from human grasping and also robotic grasping that the edges with an angle difference of up to a certain threshold can still be grasped. However, the quality and success rates of the grasp decrease with larger angles. As such, a moving threshold logic was implemented. When the algorithm is unable to find any suitable parallel pairs, it loosens the tolerance in the search for parallel edges by allowing the selected edges to have a certain difference in angles. This tolerance is increased iteratively from an initial value (e.g., 10°) to a maximum threshold (e.g., 45°) to search for a graspable pair of edges. The step size and tolerance can be defined by users. It allows the algorithm to prioritize edges of smaller angle differences over larger ones to achieve a more stable grasp if possible. Figure 11 shows an illustration of this logic.

### 2.6. Objects without Parallel Detected Edges

If a suitable pair of straight edges cannot be found after the loosening of constraints due to the shape of the object, the algorithm is equipped with additional logic to identify the general shape of such objects and hence execute a different grasping strategy. For an object with multiple features detected but none that fulfill the requirements for a parallel pair of edges, the algorithm identifies the longest straight edge based on $l_{E_{i}}$ as the point of contact for the first finger. The middle point of the edge $\left(x_{E_{i}},y_{E_{i}}\right)$ is projected at 90° towards the other side of the object and moves along the edge until it intersects the first nearest perimeter *P* at point *K* with coordinates $\left(x_{K},y_{K}\right)$. This marks the position of the second finger. The algorithm then checks the distance between these two points to ensure it is not larger than the gripper maximum opening $d_{max}$.

(10) $$\sqrt{{\left(x_{K}-x_{E_{i}}\right)}^{2}+{\left(y_{K}-y_{E_{i}}\right)}^{2}}\leq d_{max}$$

If the distance is graspable, then these two points give the grasp position. Otherwise, the algorithm discards them and moves on to the next longest edge to repeat the process until this condition is met. The grasp position is then established.

**Figure 12:**
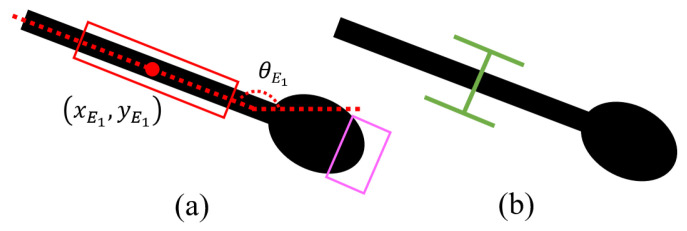
(**a**) An object with a small area and only one straight edge is detected. (**b**) The algorithm grasps at that straight edge.

If |E|=n=1 and *A* is small, the object could be a very small, stick-like object such as a spoon, as shown in Figure 12a. The algorithm grasps at position $\left(x_{E_{1}},y_{E_{1}}\right)$ and orientation $\theta_{E_{1}}$, as illustrated in Figure 12b.

### 2.7. Objects without Straight Edges

**Figure 13:**
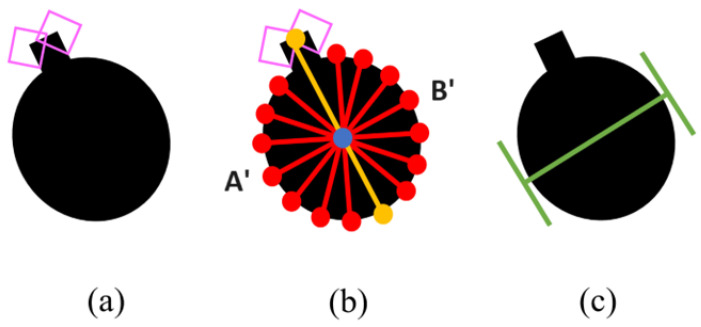
(**a**) A slight oval with 2 detected corners. (**b**) Lines are drawn through the object centroid from one end to another at varying angles. If any of these lines intersect the bounding box of any corner, these lines are eliminated. (**c**) The shortest amongst the remaining lines is chosen as the grasp position.

Objects without any straight edges (i.e., |E|=∅) could be of highly irregular shapes with straight edges too small to be detected, or circular objects. As such, the algorithm derives a grasp position using other available information. The shortest distance across the object centroid from perimeter to the other side of the perimeter would serve as a good and stable grasp position that is most likely to be within the gripper’s maximum distance. A circular object with two pointer corners is presented in Figure 13a. Multiple lines $L_{n}$, where $n_{l}$ is the number of lines, are drawn from one point $A^{\prime }$$\left(x_{A_{L_{n}}},y_{A_{L_{n}}}\right)$ along the perimeter through the object centroid to the corresponding point $B^{\prime }$$\left(x_{B_{L_{n}}},y_{B_{L_{n}}}\right)$ on the opposite side of the object perimeter, as illustrated in Figure 13b. Corners tend to be poor grasping points, as the point of contact is small, making the grasp unstable. Hence, if corners are available, a check is performed to ensure that none of the lines intersect the bounding boxes of the corners if possible. If they do, these lines are eliminated, as illustrated by the yellow line in Figure 13. The distances $D_{L_{n}}$ of the remaining lines are calculated.

(11) $$D_{L_{n}}=\sqrt{{\left(x_{A_{L_{n}}}-x_{B_{L_{n}}}\right)}^{2}+{\left(y_{A_{L_{n}}}-y_{B_{L_{n}}}\right)}^{2}}$$

The shortest line is chosen, and the grasp position can be derived as illustrated in Figure 13c.

### 2.8. Grasp Algorithm

The following pseudo-codes summarize the approaches taken by the grasping method. Algorithm 1 shows the information extraction process from an image via feature detection and image segmentation.
**Algorithm 1** Feature and parameter extraction1:Detect features *E*, *C*, form abstract shape2:Calculate $P_{e},A_{e},\left(x_{ce},y_{ce}\right)$ from abstract shape3:Image segmentation detects $P_{g},A_{g},\left(x_{cg},y_{cg}\right)$4:**if** unable to detect **then**5:    Use $P_{e},A_{e},\left(x_{ce},y_{ce}\right)$6:**else**7:    Compare $A_{g}$ and $A_{e}$ to evaluate detections

Algorithm 2 shows the steps in searching for a suitable pair of parallel straight edges closest to the object centroid.
**Algorithm 2** Search for parallel straight edges1:$d_{max}\left(px\right)=physicalmax\left(cm\right)\times \frac{focal(px)}{depth(cm)}$2:**if** n≥2 **then**3:    Calculate distance $D_{i}$ for all $E_{i}$ from $\left(x_{c},y_{c}\right)$4:    Sort $E_{i}$ by $D_{i}$ (ascending)5:    **for** $\theta_{max}=min$ to max **do**                                                ▹ user-defined min/max6:        **for** i=1 to n−1 **do**7:           **for** j=i+1 to *n* **do**8:               $\Delta \theta =|\theta_{E_{i}}-\theta_{E_{j}}|$9:               **if** $\Delta \theta \leq \theta_{max}$ **then**10:                   Draw line$d_{i}$ between $E_{i},E_{j}$11:                   Check $|\theta_{line}-\theta_{i}|\;\geq \theta_{diff\_min}$12:                   Check $d_{i}\leq d_{max}$13:                   **if** suitable **then**14:                       Break15:                   **else**16:                       j=j+117:           **if** no suitable pair **then**18:               i=i+119:        **if** no suitable pair **then**20:           $\theta_{max}=\theta_{max}+step$                                                ▹ user-defined step21:    **if** pair found **then**22:        Optimize grasp position23:    **else**24:        Sort $E_{i}$ by length $l_{E_{i}}$ (descending)25:        **for** i=1 to *n* **do**26:           *K* = Project $\left(x_{E_{i}},y_{E_{i}}\right)$ to other side of *P*27:           Distance *K* to $\left(x_{E_{i}},y_{E_{i}}\right)\leq d_{max}$28:           **if** suitable **then**29:               Break30:           **else**31:               i=i+1

Algorithm 3 shows the steps in deducing the grasp position for objects which have no suitable parallel edges for grasping.
**Algorithm 3** No parallel straight edges1:**if** n==1 and A==small **then**2:    Use $\left(x_{E_{1}},y_{E_{1}}\right)$ and $\theta_{E_{1}}$3:**else if** n==0 **then**4:    Draw lines $L_{n}$ about $\left(x_{c},y_{c}\right)$ at rotating intervals5:    Find points $A^{\prime }$ and $B^{\prime }$ intersecting *P*6:    **if** m≠0 **then**7:        **for** i = 1 to m **do**8:           Discard any $L_{n}$ if intersects $C_{i}$9:    Find $L_{n}$ with shortest length $A^{\prime }B^{\prime }$10:    Use points $A^{\prime }$, $B^{\prime }$ and $\theta_{A^{\prime }B^{\prime }}$

The full source code for the algorithm can be found here: (https://github.com/khor0054/FeatureDetectionRobotGrasping/ (accessed on 1 July 2024)).

### 2.9. Derivation of Physical Grasp Pose

The final step before carrying out the grasp action is to convert the image coordinates into the physical coordinates. Most of the algorithm logic branches give the position of two points for a two-fingered gripper to grasp. Let the coordinates of these two points be $\left(x_{1},y_{1}\right)$ and $\left(x_{2},y_{2}\right)$. The object position $\left(x_{obj},y_{obj}\right)$ and orientation $\theta_{obj}$ can be calculated from these two points for the robot to make the grasp attempt.

(12) $$\left(x_{obj},y_{obj}\right)=\left(\frac{x_{1}+x_{2}}{2},\frac{y_{1}+y_{2}}{2}\right)$$

(13) $$\theta_{obj}=\arctan\left(\frac{x_{2}-x_{1}}{y_{2}-y_{1}}\right)$$

**Figure 14:**
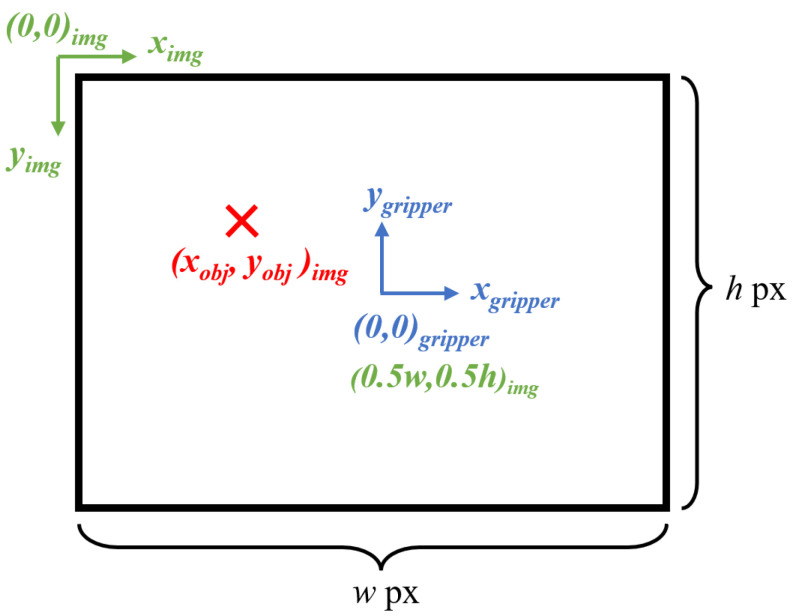
The origin of an image (green) is at the top left-hand corner, whereas the origin of the gripper (blue) is at the center of the image, which is at coordinates (0.5w,0.5h) in the image frame. For an object (red) in the image frame, adjustments are needed to convert it to the gripper frame.

Conventionally, images have their frame origin at the top left-hand corner with the *x*-axis pointing to the right and the *y*-axis downwards. However, as the camera is placed where the gripper is, its image frame origin is set at the center of the image with its *x*-axis to the right, and its *y*-axis upwards. As the images are of a fixed resolution of *w* (width) by *h* (height) pixels, the gripper’s coordinates are at (0.5w,0.5h) in the image frame. As such, further adjustments have to be made to account for this frame difference, as illustrated in Figure 14.

The coordinates of the object $\left(x_{gripper},y_{gripper}\right)$ in pixels can then be converted to the physical or real coordinates $\left(x_{real},y_{real}\right)$ in cm as follows:

(14) $$\left(x_{gripper},y_{gripper}\right)=\left(x_{obj}-0.5w,0.5h-y_{obj}\right)$$

(15) $$\left(x_{real},y_{real}\right)=\left(x_{gripper}\times \frac{d}{f},y_{gripper}\times \frac{d}{f}\right)$$

where *d* is the depth of the camera to the object in cm, and *f* is the focal length of the camera in pixels.

**Remark:** The proposed algorithm in this work is developed for two-jaw parallel grippers. The main motivation for focusing on this type of gripper is its prevalence in practical applications compared to other types, such as multi-finger grippers, vacuum grippers, and magnetic grippers. While different gripper types offer their own benefits, two-jaw grippers are the most popular for robotic manipulations due to several key advantages: versatility (capable of handling a wide range of object shapes and sizes), precision (able to generate precise gripping force when handling delicate items), simplicity and reliability (in mechanical design, control, and maintenance), operation speed (especially compared to complex multi-finger grippers), and cost-effectiveness (simpler design, fewer components, and widespread use).

## 3. Experiments

This section presents the physical experiments conducted to verify the efficiency of the proposed grasping algorithm. The details of the experimental set-ups are first presented, followed by the results of the experiments. The results and observations of the experiments are then discussed.

### 3.1. Experimental Setup

**Figure 15:**
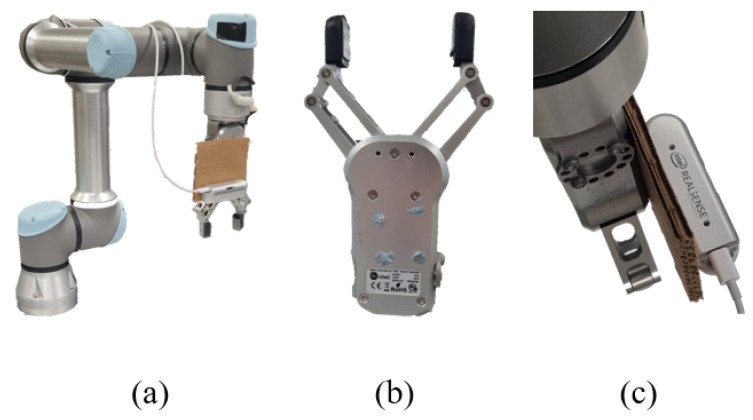
From left to right: (**a**) arm, (**b**) gripper, and (**c**) camera.

In the experiments, a total of 400 images of around 100 different objects from the Cornell Grasping Dataset were used as the training data. Only the images from the dataset were used; the other grasping information was discarded. Any oriented object detector can be used, but for this study, a modified version of You-Only-Look-Once version 5 (YOLOv5) algorithm called YOLOv5 OBB (Oriented Bounding Boxes) [23] was chosen as the choice of oriented object detector. Training was conducted on a Linux server equipped with a 32 GB Tesla V100-DGXS Graphics Processing Unit (GPU) over 1000 epochs, taking around 10 h to complete. The robot arm used is a Universal Robot UR5e equipped with an OnRobot RG2 Robot Gripper and the camera attached is an Intel RealSense. Both the robot and camera are connected to a PC running a 64-bit Ubuntu 18.04.5 LTS Operating System with an Intel Core i5-7500 CPU (Intel Corporation, Santa Clara, CA, USA) and a NVIDIA GeForce RTX 2080 SUPER GPU (NVIDIA, Santa Clara, CA, USA). A white background is used for the object to be grasped. For each object tested, 10 attempts were made. In total, 40 different objects were used for a total of 400 grasp attempts. In the 10 attempts of each object, the position and orientation of the object are also varied within the field of view of the camera. The tactile sensors in the robot’s gripper are set to a fixed threshold. Once the gripper closes in on the object and detects the threshold force, it assumes that the object is already grasped and stops closing the gripper further. The arm then proceeds to lift the object up. The robot commands are executed based on the inner controller of UR5e with closed architecture and no external feedback controller is required. A grasp is considered successful if the robot is able to grasp it and return it to its ending position without dropping it in the moving process. The hardware used is shown in Figure 15.

### 3.2. Object Test Sets

**Figure 16:**
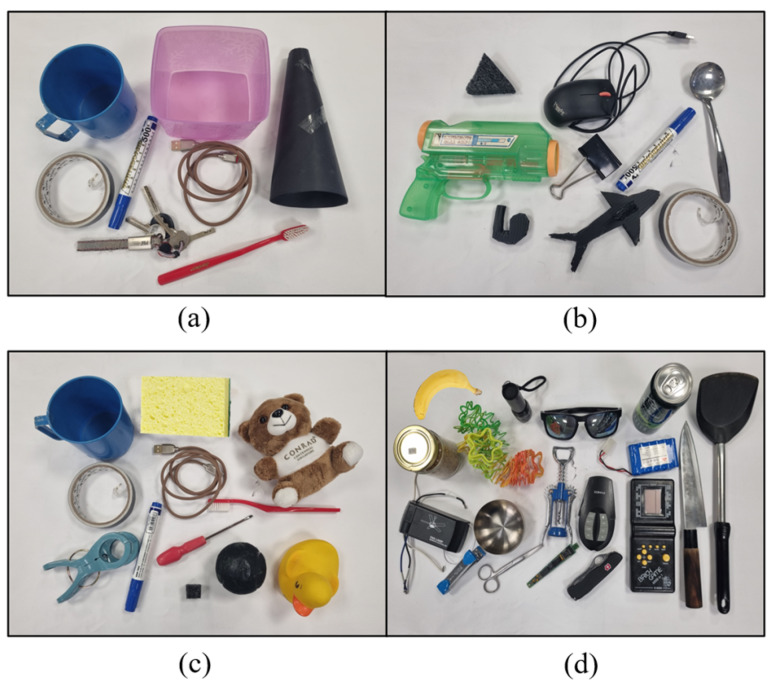
(**a**) Similar object set used for GPP testing [18]. (**b**) Similar object set used for DexNet 2.0 testing [19]. (**c**) Similar object set used for GG-CNN testing [20]. (**d**) Object set designed by this study for algorithm testing.

Four different sets of test objects were used. The first three sets of objects closely replicate the objects used in the evaluation of the following grasping algorithms in the literature: Grasp Point Prediction (GPP) [18], DexNet 2.0 [19], and GG-CNN [20]. This allows for a point of comparison. The fourth set of objects is a self-designed set of objects to illustrate the capabilities of the algorithm in a wide variety of scenarios. Figure 16 shows all these four sets of objects used for testing.

Figure 16a shows the objects tested by GPP [18], containing eight objects, with each grasped four times. The objects are tape, keys, marker, toothbrush, mug, translucent box, horn, and coiled wire. Figure 16b shows the objects tested by DexNet 2.0 [19], containing nine objects, with each grasped five times. The objects are spoon, marker, toy shark, clip, whistle, tape, mouse, glue gun, and a black triangular object. Figure 16c shows the objects tested by GG-CNN [20], containing 12 objects, with each grasped 10 times. The objects are mug, tape, clothes peg, sponge, coiled wire, marker, stuffed toy, toothbrush, screwdriver, die, ball, and rubber duck. To further illustrate the abilities of the proposed algorithm, 18 other objects, as shown in Figure 16d, were tested on top of those used by the three studies. These objects are stapler, scissors, glasses, flashlight, banana, bowl, canned drink, Swiss army knife, kitchen knife, circuit board, wine opener, remote control, spatula, battery pack, game console, jar, electrical component, and a slinky toy.

### 3.3. Results

**Figure 17:**
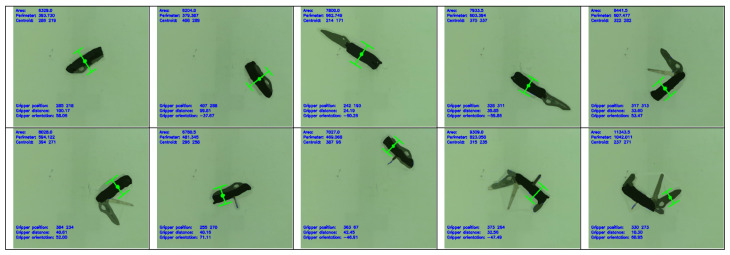
Grasp positions for 10 grasp attempts on a Swiss Army Knife with varying positions, orientations, and tool configurations.

The proposed method is able to generate a grasp position for each object in 0.1 s. Figure 17 shows an example of the results of a typical experiment for an object. In this case, the object is a Swiss Army Knife. The figure shows how the position and orientation of the object are varied across all grasp attempts. Where possible, the shape of the object is also manipulated across attempts. In this example, the various tools of the Swiss Army Knife are opened in different combinations.

**Figure 18:**
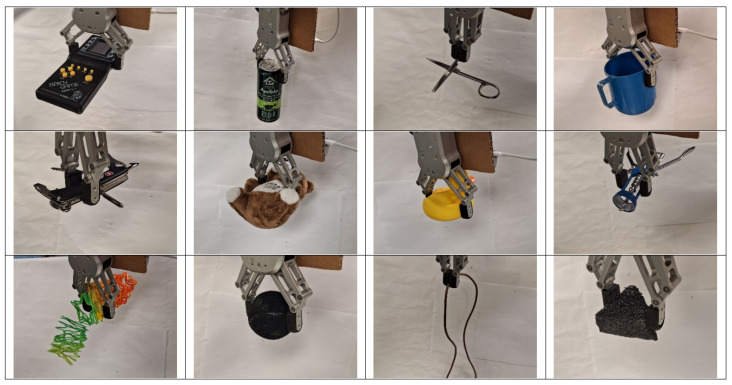
Successful grasps for game console, canned drink, scissors, stuffed toy, Swiss Army Knife, rubber duck, mug, wine opener, slinky toy, ball, wire, and triangle.

**Figure 19:**
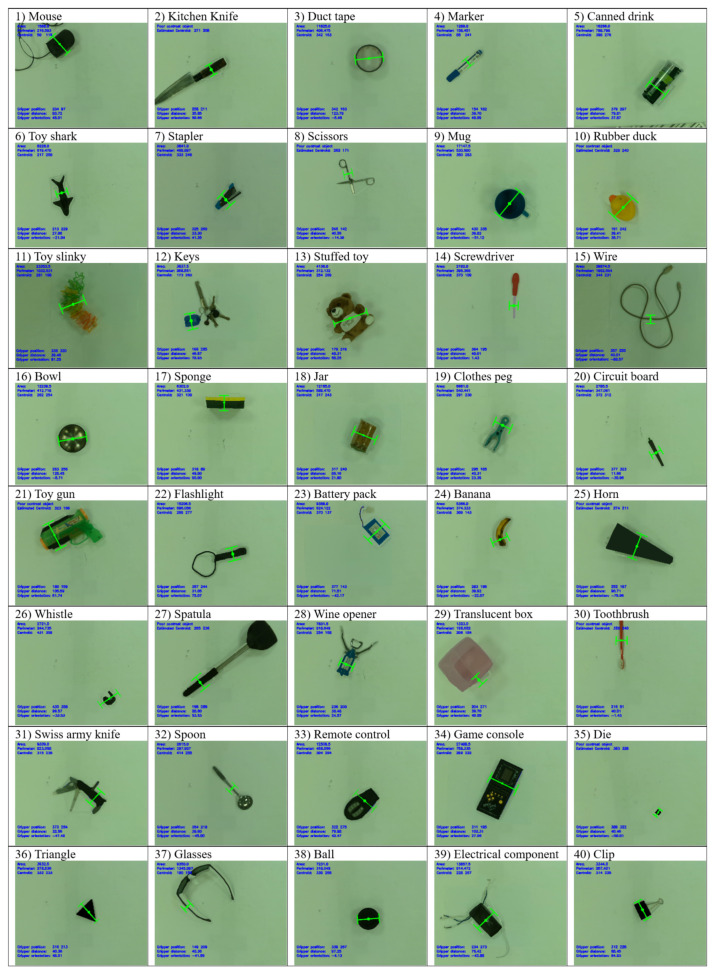
Examples of grasp poses for each of the 40 objects across all test sets generated by the algorithm.

Table 1 summarizes the statistics for the three existing methods that were used for comparison. The proposed methods were tested on a total of 40 different objects, with 10 attempts for each object, yielding a total of 400 grasp attempts. Table 2 summarizes the grasp success rate of the proposed method on the four different object test sets, as well as the total success rate. Some objects are repeated across the three test sets [18,19,20] (duct tape is present in all three studies but is only counted once in the total success rate). Table 3 shows the full breakdown of all the items used and the success rate of each of them. Figure 18 shows a few examples of the physical grasp on the object by the robot after returning to its ending position and Figure 19 shows an example of a grasp position generated by the algorithm for each object tested.

### 3.4. Discussion

The proposed algorithm demonstrates a superior ability to grasp a large variety of unseen objects. Rectangular-shaped objects like the game console (Figure 19, Object 34) are easily graspable due to their obvious straight edges. Even if they have some stray components like loose wires found in the battery pack (Figure 19, Object 23) and the electrical component (Figure 19, Object 39), the algorithm is able to ignore them and still grasp along the straight edges. Some items with smaller straight edges like the set of keys (Figure 19, Object 12) and the die (Figure 19, Object 35) are also identifiable by the algorithm. It is worth noting that the algorithm works particularly well with handled objects like the kitchen knife (Figure 19, Object 2) and the spatula (Figure 19, Object 27), attributable to the property that handles are usually straight and thick, which makes for very prominent parallel edges. Other odd-shaped objects like the Swiss Army Knife (Figure 19, Object 31) and the wine opener (Figure 19, Object 28) are also graspable, as they have obvious parallel edges despite the overall irregular shape of the object.

**Figure 20:**
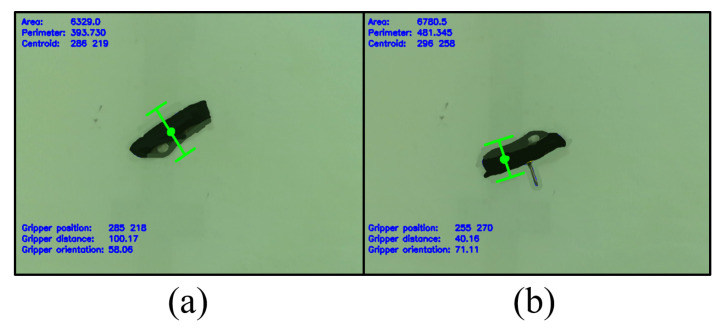
(**a**) When closed or with just the main blade open, the algorithm targets the middle of the handle for grasping. (**b**) With the corkscrew tool open at the middle, the algorithm shifts the grasp position to the sides of the handle to avoid collision with the tool.

The Swiss Army Knife, in particular, showcases the advantage of the algorithm in terms of flexibility. Being a highly transformable object, the Swiss Army Knife was changed in shape significantly over the course of testing, as illustrated in Figure 17. The algorithm aims for the middle of the handle, as in Figure 20a, where possible. When the corkscrew tool is opened at the middle of the handle, the algorithm automatically shifts its grip to the side of the Swiss Army Knife to avoid collision with the corkscrew tool in Figure 20b.

The translucent box (Figure 19, Object 29) showcases the maximum gripper distance logic of the algorithm. Being a square box, it has obvious straight edges. However, this box was deliberately chosen, as it has a length of about 12 cm compared to the gripper’s maximum opening of 10 cm. The algorithm is able to determine that it is too wide for grasping and hence attempts to pick up the box by one edge instead.

The horn (Figure 19, Object 25) and the remote control (Figure 19, Object 33) are examples of objects that do not have perfectly parallel edges. However, they are still graspable due to the increasing threshold logic of the algorithm. The triangle (Figure 19, Object 36) is grasped by projecting one edge to the other side of the object perimeter, which happens to be a corner. With one edge and one corner in contact, the robot is able to grasp it.

Small, stick-like objects that have only one straight edge detected, like the screwdriver (Figure 19, Object 14) and the toothbrush (Figure 19, Object 30), are grasped along this edge. For other odd-shaped objects like scissors (Figure 19, Object 8) and glasses (Figure 19, Object 37), the algorithm is also able to identify a straight edge to grasp at.

The algorithm is unable to identify any straight edges on objects like the mug (Figure 19, Object 9), the rubber duck (Figure 19, Object 10), and the ball (Figure 19, Object 38). Some have corners detected, like the handle of the mug and the bill of the rubber duck, while some are circular with absolutely no detected features at all, like the ball and duct tape (Figure 19, Object 3). The algorithm is able to derive a stable grasp about the object centroid. Avoiding the corners contributes to the stability of the grasps as well. The rubber duck had two detected corners at the beak and the tail. These would likely make for very small contact points with the gripper and result in a less stable grasp. The object which the algorithm had the most difficulty with is the toy slinky (Figure 19, Object 36). Due to the highly irregular shape of the object, the algorithm had difficulty in identifying clear features of the object. Moreover, the malleable nature of the object made it challenging for the robot to grasp, as it tends to change its shape and move out of the intended grasp position. However, in spite of these challenges, the algorithm is still able to execute a stable grasp by targeting the approximate centroid of the object. This allows the object to be more balanced and less likely to slide out of the gripper. Hence, the algorithm still sports a high success rate in grasping the toy slinky, further showing its flexibility in dealing with challenging situations.

The algorithm sports a significantly higher grasp success rate compared to other existing methods on a significantly smaller training dataset. This can be attributed to the greatest advantage of this algorithm, which is flexibility. In other existing methods, when encountering an object, the algorithm finds the closest match based on what it is trained on. However, as illustrated in Figure 17, even within a single object, there can be many orientations, positions, and configurations. This makes it impossible to train an algorithm to be able to deal with every possible combination. In contrast, the features of an object do not change as easily. Even when changing configurations, the key features of the object remain unchanged. The algorithm is equipped to reactively deduce a grasp position based on the features presented, providing it with a high level of flexibility. Unlike other methods where the grasp position of an object in every orientation and configuration has to be labelled, this study has demonstrated that with feature detection, this tedious labelling process is not required and the algorithm has the flexibility to handle a large variety of cases.

## 4. Conclusions

In this paper, a simple and effective algorithm has been proposed to enable robots to grasp unknown objects. The proposed algorithm resorts to an oriented object detector (YOLOv5 OBB in this work) to identify two common key features present in most objects, namely straight edges and corners, instead of complex object classes. Meanwhile, the object geometric parameters can be obtained using an image segmentation algorithm. Based on the identified features and geometric information obtained from image segmentation, the algorithm can deduce information about the object and accordingly derive suitable grasp positions following predefined logic rules. Experimental studies have been carried out to grasp 40 different unknown objects, and the experiment results show that the algorithm can achieve a higher success rate of 98.25 % in unknown object grasping than existing methods.

The proposed method has achieved a high success rate using only geometric information of the object. Further enhancements can be made by detecting other features, such as surface roughness and object rigidity, which may improve grasp performance. Soft objects tend to deform and exert much lower normal forces on sensors compared to rigid objects, increasing the likelihood of slippage. Therefore, if the applied force is too low, the grasp is likely to be inadequate. By utilizing tactile sensors and incorporating additional object information, these factors can be partially addressed.

## Figures and Tables

**Table 1:** Grasp success rates of existing methods.

Methods	Training	Attempts	Success
	**Size**		**Rate (%)**
GPP [18]	2500	32	87.50
DexNet2.0 [19]	6.7 million	45	80.00
GG-CNN [20]	51,100	120	92.00

**Table 2:** Grasp success rates of proposed method on various object test sets.

Method	Training	Test Set	Attempts	Success
	**Size**			**Rate (%)**
Proposed method	400	similar to GPP	80	97.50
		similar to DexNet2.0	90	97.78
		similar to GG-CNN	120	99.17
		Self-designed	180	98.33
		All objects	400	98.25

**Table 3:** Grasp success rate of each object for 10 trials each.

Object	Success Rate (%)	Object	Success Rate (%)
Mouse	100	Kitchen knife	100
Duct tape	100	Marker	100
Canned drink	100	Toy shark	100
Stapler	100	Scissors	100
Mug	100	Rubber duck	100
Slinky toy	90	Keys	90
Stuffed toy	100	Screwdriver	100
Wire	90	Bowl	100
Sponge	100	Jar	90
Clothes peg	100	Circuit board	100
Toy Gun	90	Flashlight	100
Battery pack	100	Banana	100
Horn	100	Whistle	100
Spatula	100	Wine opener	100
Translucent box	100	Toothbrush	100
Swiss army knife	100	Spoon	90
Remote Control	100	Game console	100
Die	100	Triangle	100
Glasses	90	Ball	100
Electrical component	100	Clip	100
**Overall success rate:** 98.25%			

## Data Availability

Data are contained within the article.
